# Social isolation, loneliness and physical performance in older-adults: fixed effects analyses of a cohort study

**DOI:** 10.1038/s41598-020-70483-3

**Published:** 2020-08-17

**Authors:** Keir E. J. Philip, Michael I. Polkey, Nicholas S. Hopkinson, Andrew Steptoe, Daisy Fancourt

**Affiliations:** 1 NHLI Respiratory Muscle Laboratory, National Heart and Lung Institute, Imperial College London, Royal Brompton Campus, Fulham Rd., London, SW3 6NP UK; 2 Respiratory Medicine, Royal Brompton and Harefield NHS Foundation Trust, London, UK; 3 Department of Behavioural Science and Health, University College London, London, UK

**Keywords:** Geriatrics, Public health, Quality of life

## Abstract

Isolation and loneliness are related to various aspects of health. Physical performance is a central component of health. However, its relationship with isolation and loneliness is not well understood. We therefore assessed the relationship between loneliness, different aspects of social isolation, and physical performance over time. 8,780 participants from the English Longitudinal Study of Ageing, assessed three times over 8 years of follow-up, were included. Measures included physical performance (Short Physical Performance Battery), loneliness (modified UCLA Loneliness Scale), and isolation considered in three ways (domestic isolation, social disengagement, low social contact). Fixed effects regression models were used to estimate the relationship between changes in these parameters. Missing data were imputed to account for variable response and ensure a representative sample. Loneliness, domestic isolation and social disengagement were longitudinally associated with poorer physical performance when accounting for both time-invariant and time-variant confounders (loneliness: coef = − 0.06, 95% CI − 0.09 to − 0.02; domestic isolation: coef = − 0.32, 95% CI − 0.46 to − 0.19; social disengagement: coef = − 0.10, 95% CI − 0.12 to − 0.07). Low social contact was not associated with physical performance. These findings suggest social participation and subjectively meaningful interpersonal interactions are related to physical performance, and highlight additional considerations regarding social distancing related to COVID-19 control measures.

## Introduction

Social factors including isolation (frequency of social interactions) and loneliness (the subjective quality of social interaction) are related but distinct concepts, both of which have been shown to be associated with morbidity and mortality^1,2^. Much of this literature has focused on mental health, showing associations with higher levels of stress and depression^3^, but there has been increasing interest in effects on physical health. For example, both isolation and loneliness have been linked with systemic inflammation^4,5^, autonomic dysfunction^6,7^, neuroendocrine dysregulation^8^, chronically increased allostatic load^9^, stroke and coronary heart disease^10^.

However, a less researched area is the relationship between changes in isolation, loneliness and physical performance. Physical performance, based on objective testing, depends on factors including strength, balance and endurance is a key component of physical function, mobility and independence^11,12^. Impaired physical performance can be associated with significant personal, societal and economic costs^13^. Furthermore, understanding the relationship between social isolation, loneliness and physical performance in older-adults has become acutely important in the context of the COVID-19 pandemic. Because older people are particularly at risk from the disease, social distancing measures have been introduced throughout the world to reduce their exposure to SARS-CoV-2, the virus that causes COVID-19. These measures are appropriate in the current context but are likely to have various unintended mental and physical health impacts. Improving our understanding of these impacts can facilitate mitigation of negative sequalae through targeted interventions, such as homebased exercise programmes and online communal activities.

A small number of studies have suggested relationships between social isolation, loneliness and physical performance. For example, loneliness and less frequent engagement in social activities have been associated with reductions in objective markers of motor function in older adults^14,15^ and walking speed^16^. Other studies using subjective measures of function have shown that individuals with more social ties show slower rates of functional decline^17^, and reduced participation in social activities has been linked to functional disability^18^. However, these studies have focused on how social factors measured at a single point in time are associated with physical performance in subsequent years. Yet social isolation and loneliness are dynamic states that fluctuate, both according to life circumstances but also as people age. Further, there are a wide range of potential confounding factors that also vary over time and could explain any potential association. Additionally, isolation can, and has been, defined in a variety of ways. People may be isolated because they live alone, because they have few social interactions with friends or family, or because they don’t participate in organisations and community activities. The inclusion or exclusion of various components of isolation may influence the relationship between these variables and physical performance. This is of crucial importance if the research findings are to make useful contributions to planning health and social care interventions and policy.

To address these challenges, we used a statistical approach that enables the tracking of patterns of behaviour over time to explore the relationship between time-varying loneliness and social isolation and time-varying physical performance amongst a representative sample of adults aged 50+ in England, whilst accounting for time-varying confounding demographic and life-style factors. In addition, in this study we also explored three different types of social isolation to help clarify which components of isolation were of greatest importance in relation to physical performance.

## Methods

### Study design and participants

We used data from the English Longitudinal Study of Ageing (ELSA), a nationally representative cohort study of community dwelling adults aged over 50 in England. The sample was drawn from households that had previously responded to the Health Survey for England (HSE) in 1998, 1999 or 2001. HSE used a two-stage sampling design consisting of the selection of postcode sectors from a postcode address file and then subsequent selection of postcode addresses from within each postcode sector^19^. All core participants from wave 2 of ELSA (2004/05—when physical performance was first measured) were included (n = 8,780), and were followed up in waves 4 (2008/09) and 6 (2012/13). We maintained a sample size of 8,780 participants through the waves by imputation of missing data, as described below. In ELSA, the primary method of data collection includes computer assisted interviews which are completed face-to-face at the participants’ usual place of residence, and a physical assessment conducted by a qualified nurse trained in the specific study protocols. Full documentation of data collection protocols is available at https://www.elsa-project.ac.uk/. ELSA received ethical approval from the National Research Ethics Service and all participants provided written informed consent.

### Measures

#### Physical performance

Physical performance was assessed using the Short Physical Performance Battery (SPPB), which is rated highly in terms of validity, reliability and responsiveness^20^. SPPB assesses three components: five times sit-to-stand, standing balance, and gait-speed^21^. Sit-to-stand was assessed by timing participants completing five repetitions of standing from a seated position, without using their arms to push themselves up. Balance was assessed through sequentially more difficult balance tasks involving standing first with feet side-by-side, then with feet in semi-tandem, then in full tandem. Participants were asked to try and stay in each position for 10 s without moving their feet or holding any supports, with their ability to complete the task scored according to established categories. Walking speed was assessed by timing participants as they completed an 8ft (2.44 m) walking course at their normal speed, with the fastest speed of two attempts recorded. Walking speed was only measured for those over the age of 60, so for those below we used multiple imputation to impute missing values and then ran sensitivity analyses for individuals above and below the age of 60 to assess whether the results were affected by these imputations. Each component was then categorised into a score ranging from 0 to 4, with the scores for each of these three components then summed to provide a total score from 0 to 12 (with higher scores indicating better function), see Guralnik et al. for further details regarding scorin^21^. A detailed standardised protocol for the performance tests is available online at https://www.elsa-project.ac.uk/data-and-documentation.

#### Social isolation

We used three measures of social isolation. Domestic isolation we defined based on whether individuals lived alone or not. Although domestic isolation is highly correlated with marital status, being married does not guarantee living with a spouse, and individuals who are unmarried can live with friends or family. So, living status is a more reliable marker of domestic isolation.

To assess social contact, we used self-reported frequency of social interactions. This included (a) face to face interaction, (b) telephone conversations, or (c) email or written communication with (1) children, (2) other family members or (3) friends. A point was given for use of each mode of communication with each group of people that an individual did not have contact with, providing an overall score from 0 to 9 with higher scores indicating greater social isolation. The index had a Cronbach’s alpha of 0.80.

For social disengagement, we measured frequency of (1) participation in community group activities (including political party, trade union or environmental groups, tenant groups, resident groups, neighbourhood watch groups, church or other religious groups, charitable associations, education, arts or music groups or evening classes, social clubs, sports clubs, exercise classes, or any other organisations, clubs or societies), and (2) engagement with community cultural activities (including going to museums, exhibitions, the theatre, concerts, opera or the cinema). Frequency of community group activities was measured as number in the past 12 months and then recoded as never (score of 4), once or twice a year (score of 3), every few months (score of 2), or monthly or more (score of 1). Frequency of cultural activities was as never (score of 4), less than once a year (score of 3), once or twice a year (score of 2), or every few months or more (score of 1). These scores were summed to provide an overall index of 2–8, with higher scores indicating higher levels of social disengagement. The index had a Cronbach’s alpha of 0.73.

#### Loneliness

Loneliness was measured using an adapted 3-item questionnaire^22^ based on the UCLA loneliness scale, which has strong psychometric properties^23^. ELSA respondents were asked how often they (1) felt that they lacked companionship (2) felt left out and (3) felt isolated from the people around them. Frequencies ranged from hardly ever or never (assigned a score of 3) some of the time (assigned a score of 2) and often (assigned a score of 1). The scores for each measure were then summed to give a loneliness score ranging from 3 to 9 where higher scores indicated higher levels of loneliness. The index had a Cronbach’s alpha of 0.83.

#### Covariates

Time variable covariates included age (continuous), marital status (married/cohabiting vs other), employment status (working part-time or full-time vs not currently working), wealth (quintiles). Health related covariates included body mass index (BMI) (continuous), eyesight (very poor/registered blind vs fair/good/very good/excellent); presence of significant physician diagnosed co-morbidities (binary composite variable including arthritis, Parkinson’s disease, congestive heart failure, ‘other heart problem’, Alzheimer’s disease or other dementia, previous stroke with ongoing limb weakness, chronic lung disease such as chronic bronchitis, asthma); chronic pain (no/mild vs moderate/severe); frequency of alcohol consumption (less than once a week vs once to four times per week vs five or more times per week); smoking (never vs ex-smoker vs current); inactive (binary variable—undertakes any kind of sports or activities that are mildly energetic less than weekly vs at least weekly); cognition (using an index of scores from neuropsychiatric batteries testing memory, executive function, processing speed and orientation in time, averaged and standardised); and depression (using the Centre for Epidemiological Studies scale CES-D^24^).

### Statistical analysis

Analyses were carried out using Stata 14 (StataCorp, College Station, TX). Missing data were imputed using multiple imputation by chained equations to provide 50 imputed datasets using the following predictor variables: physical performance (total SPPB score, five times sit-to-stand score, balance and walking speed), demographic factors (age, sex, marital status, employment status and wealth), and health factors (BMI, eyesight, comorbidities, chronic pain, frequency of alcohol consumption, smoking habits, inactivity, cognition and mental health). Patterns of missing data are shown in the Supplementary Material (Supplementary Table 9).

Fixed effects regression models were used to estimate the relationship between changes in isolation, loneliness and physical performance. Fixed effects regression is a longitudinal statistical technique that has several strengths: (1) it considers time-varying relationships, which is important when considering states such as isolation and loneliness which are likely to change as people age and be influenced by other time-varying factors such as health or retirement status; (2) in fixed-effects regression, within-person variation is explored with individuals acting as their own reference point over time. So all time-invariant factors (e.g. gender, ethnicity, genetics, personality, educational attainment and socio-economic status) are accounted for even if unobserved^25^. This can help to reduce the potential for unobserved confounding leading to spurious results. The basic model for physical performance (SPPB) can be expressed as follows:

$${SPPB}_{it}={{\beta }_{0t}+ {\beta }_{1}S}_{it}+ {\beta }_{2}{T}_{it}+{\alpha }_{i}+ {\varepsilon }_{it}$$

where SPPB_it_ is a measure of individual i’s levels of physical performance at time t, α_i_ is unobserved time invariant confounding factors, S is whether an individual was experiencing isolation or loneliness, at time t, and T is measured time-varying confounding factors. Data were strongly balanced. A Hausman test was used to confirm the selection of a fixed effects over a random effects model. The modified Wald test for group-wise heteroscedasticity was significant so sandwich estimators were applied. Coefficients for all years were not jointly equal to zero, so time-fixed effects were included in the model. All other model assumptions were met.

In addition to time-invariant factors already considered in the model and time itself (model 1), model 2 additionally adjusted for time-varying demographic factors (age, marital status, employment status and wealth). Model 3: additionally adjusted for time-varying health factors (BMI, eyesight, comorbidities, chronic pain, frequency of alcohol consumption, smoking habits, inactivity and cognition). Model 4: additionally adjusted for time-varying mental health (depression).

Our main analyses involved entering all exposures simultaneously in the model. But our first sensitivity analysis tested the consistency of results when running separate models for each exposure. Other sensitivity analyses included (1) additional measures of moderate and vigorous physical activity alongside the measure of inactivity to confirm that time-varying engagement in sports or energetic activities did not act as a confounding factor; (2) responses split by gender and age (above and below 60 and 65) to identify if any relationship was more clearly present in some groups than others; (3) a binary outcome variable for SPPB categorising responses into above or below a score of 10, which indicates increased risk of disability^26^ and all-cause mortality^27^. All analyses were weighted using cross-sectional sampling weights.

## Results

### Descriptive analysis

Of the 8,780 participants, 53.9% were female, the average age was 69.0 years (standard error = 0.07). Of these, 47.0% had either no or only basic qualifications (less than GCSE/O-level/qualification at age 16). At baseline, a quarter of the sample (25.5%) were employed in either full time or part time work. Full details of the sample are provided in Table 1. Domestic isolation was associated only very slightly with low social contact (r = 0.10, p < 0.001, 1.0% of variance shared) and greater social disengagement (r = 0.09, p < 0.001, 0.8% of variance shared) and showed a small association with higher loneliness (r = 0.27, p < 0.001, 7.2% of variance shared). Low social contact was associated in a very small way with greater social disengagement (r = 0.06, p < 0.001, 0.4% of variance shared) and loneliness (r = 0.08, p < 0.001, 0.6% of variance shared) (Supplementary Table 8). Social disengagement and loneliness were very slightly associated (r = 0.18, p < 0.001, 3.2% of variance shared). Over time, there was a slight decline in social activities and physical function (see Supplementary Table 1, which also shows within and between variation in the standard deviation of measures).

**Table 1:** Weighted participant characteristics at baseline.

Time-invariant characteristics (stated at baseline)^a^		
Gender	% female	53.9%
Ethnicity	White (%)	97.1%
Educational attainment^b^	No qualifications/basic qualifications	47.0%
	GCSE/O-level/qualification at age 16	15.9%
	A-levels/higher education/qualification at age 18	25.9%
	Degree/further higher qualification	11.2%
Time-varying characteristics (stated at baseline)		
Age	Mean (standard error)	69.0 (0.07)
Employment	Working full- or part-time (%)	25.5%
Wealth	Split into quintiles	–
BMI	Mean (standard error)	26.4 (0.07)
Eyesight	% with very poor eyesight/registered blind	4.0%
Chronic health conditions	% with one or more of cancer, chronic lung conditions, arthritis, stroke, diabetes, angina	45.5%
Chronic pain	% with moderate or severe chronic pain	27.9%
Alcohol consumption	Less than once a week	41.1%
	Once to four times a week	36.5%
	5 or more times a week	22.4%
Smoking status	Never smoked (%)	36.5%
	Ex-smoker (%)	53.3%
	Current smoker (%)	10.2%
Inactivity	Undertakes any kind of sports or energetic activities less than weekly (%)	8.8%
Cognition	Standardised	–
Depression	(scored 3 + in CES-D) (%)	21.3%

^a^Excluded from the analysis as time-invariant factors are automatically included within fixed-effects models, but shown here for descriptive purposes.

^b^Considered to be time-invariant for the purpose of this investigation and therefore automatically included within analyses.

### Social isolation

Domestic isolation and social disengagement were longitudinally associated with poorer physical performance. Although time-varying demographic and health-related factors explained some of this association, results were significant even when accounting for all time-invariant and time-variant confounders (domestic isolation: coef = − 0.32, 95% CI − 0.46 to − 0.19; social disengagement: coef = − 0.10, 95% CI − 0.12 to − 0.07) (see Table 2). When exploring the subscales of physical performance, social disengagement was associated with lower ability in sit-to-stand, poorer balance and slower walking speed, but domestic isolation was only associated with poorer balance and walking speed. In terms of effect size, the change from living alone to living with somebody was associated with an increase in average SPPB score from 9.59 (95% CI 9.51–9.67) to 9.94 (95% CI 9.91–9.97); an improvement of 4.0%. The change from being socially completely disengaged to highly engaged, was associated with an increase in average SPPB score from 9.72 (95% CI 9.66–9.77) to 10.05 (95% CI 9.99–10.12); an improvement of (3.5%). Low social contact was not associated with poorer physical performance or any of its subscales.

**Table 2:** Results from fixed effects models showing the relationship between isolation, loneliness and physical performance: all predictors entered simultaneously.

	Total physical performance		5 times sit-to-stand		Balance		Walking speed	
	Coef (95% CI)	p	Coef (95% CI)	p	Coef (95% CI)	p	Coef (95% CI)	p
Domestic isolation								
Model 1	− 1.454 (− 1.71 to − 1.37)	< 0.001	**− 0.47 (− 0.56 to − 0.39)**	**< 0.001**	− **0.49 (**− **0.56 to **− **0.43)**	**< 0.001**	**− 0.57 (− 0.64 to − 0.50)**	**< 0.001**
Model 2	**− 0.36 (− 0.50 to − 0.21)**	**< 0.001**	− 0.05 (− 0.13 to 0.02)	0.16	**− 0.15 (− 0.21 to − 0.08)**	**< 0.001**	**− 0.16 (− 0.22 to − 0.09)**	**< 0.001**
Model 3	**− 0.34 (− 0.48 to − 0.21)**	**< 0.001**	− 0.05 (− 0.13 to 0.02)	0.15	**− 0.14 (− 0.20 to − 0.08)**	**< 0.001**	**− 0.15 (− 0.21 to − 0.09)**	**< 0.001**
Model 4	**− 0.32 (− 0.46 to − 0.19)**	**< 0.001**	− 0.05 (− 0.12 to 0.03)	0.20	**− 0.14 (− 0.20 to − 0.07)**	**< 0.001**	**− 0.14 (− 0.20 to − 0.08)**	**< 0.001**
Low social contact								
Model 1	0.009 (− 0.04 to 0.02)	0.54	− 0.007 (− 0.02 to 0.01)	0.39	− 0.004 (− 0.01 to 0.01)	0.47	0.002 (− 0.01 to 0.02)	0.81
Model 2	0.004 (− 0.03 to 0.02)	0.77	− 0.005 (− 0.02 to 0.01)	0.49	− 0.001 (− 0.01 to 0.01)	0.80	0.002 (− 0.01 to − 0.02)	0.69
Model 3	0.002 (− 0.02 to 0.03)	0.90	− 0.004 (− 0.02 to 0.01)	0.55	0.001 (− 0.01 to 0.01)	0.88	0.005 (− 0.007 to 0.02)	0.39
Model 4	0.001 (− 0.02 to 0.03)	0.94	− 0.005 (− 0.02 to 0.01)	0.53	0.001 (− 0.01 to 0.01)	0.90	0.005 (− 0.007 to 0.02)	0.42
Social disengagement								
Model 1	**− 0.27 (− 0.30 to − 0.23)**	**< 0.001**	**− 0.08 (− 0.09 to − 0.06)**	**< 0.001**	**− 0.08 (− 0.10 to − 0.07)**	**< 0.001**	**− 0.11 (− 0.12 to − 0.09)**	**< 0.001**
Model 2	**− 0.14 (− 0.17 to − 0.11)**	**< 0.001**	**− 0.03 (− 0.05 to − 0.02)**	**< 0.001**	**− 0.05 (− 0.06 to − 0.03)**	**< 0.001**	**− 0.06 (− 0.08 to − 0.05)**	**< 0.001**
Model 3	**− 0.10 (− 0.13 to − 0.07)**	**< 0.001**	**− 0.02 (− 0.04 to − 0.01)**	**0.003**	**− 0.03 (− 0.05 to − 0.02)**	**< 0.001**	**− 0.04 (− 0.06 to − 0.03)**	**< 0.001**
Model 4	**− 0.10 (− 0.12 to − 0.07)**	**< 0.001**	**− 0.02 (− 0.04 to − 0.01)**	**0.004**	**− 0.03 (− 0.05 to − 0.02)**	**< 0.001**	**− 0.04 (− 0.06 to − 0.03)**	**< 0.001**
Loneliness								
Model 1	**− 0.16 (− 0.20 to − 0.12)**	**< 0.001**	**− 0.06 (− 0.08 to − 0.04)**	**< 0.001**	**− 0.03 (− 0.05 to − 0.02)**	**< 0.001**	**− 0.07 (− 0.09 to − 0.05)**	**< 0.001**
Model 2	**− 0.14 (− 0.17 to − 0.11)**	**< 0.001**	**− 0.05 (− 0.07 to − 0.03)**	**< 0.001**	**− 0.03 (− 0.04 to − 0.02)**	**< 0.001**	**− 0.06 (− 0.07 to − 0.05)**	**< 0.001**
Model 3	**− 0.09 (− 0.12 to − 0.06)**	**< 0.001**	**− 0.03 (− 0.05 to − 0.02)**	**< 0.001**	**− 0.02 (− 0.03 to − 0.002)**	**0.02**	**− 0.04 (− 0.05 to − 0.02)**	**< 0.001**
Model 4	**− 0.06 (− 0.09 to − 0.02)**	**0.001**	**− 0.03 (− 0.04 to − 0.01)**	**0.002**	− 0.01 (− 0.02 to 0.01)	0.23	**− 0.02 (− 0.03 to − 0.01)**	**0.002**

N = 8,780, 3 observations per person, total observations 26,340. Model 1: accounting for all time-invariant factors and time. Model 2: additionally adjusted for time-varying demographic factors (age, marital status, employment status and wealth). Model 3: additionally adjusted for time-varying health factors (BMI, eyesight, comorbidities, chronic pain, frequency of alcohol consumption, smoking habits, inactivity and cognition). Model 4: additionally adjusted for time-varying mental health (depression).

### Loneliness

Higher levels of loneliness were also longitudinally associated with lower physical performance. Although time-varying demographic and health-related factors explained some of this association, results were significant even when accounting for all time-invariant and time-variant confounders (coef = − 0.06, 95% CI − 0.09 to − 0.02) (see Table 2). When exploring the subscales of physical performance, there was a significant association with poorer sit-to-stand and slower walking speed but not poorer balance, for which the association was attenuated when accounting for depression (coef = − 0.01, 95% CI − 0.02 to 0.01). If an individual improved from having the highest possible loneliness score to the lowest possible loneliness score, this was associated with an increase in their average SPPB score 9.73 (95% CI 9.62–9.85) to 9.90 (95% CI 9.87–9.93); a 1.7% improvement.

### Sensitivity analyses

When incorporating our exposures separately into the models so they did not mutually adjust for one another, the pattern of results was completely maintained, with the exception that loneliness was still related to balance when not adjusting for isolation (see Supplementary Table 2). When additionally adjusting for both moderate and vigorous sports or energetic activities, results were materially unaffected (see Supplementary Table 3). When splitting results by gender, findings were almost identical (see Supplementary Table 4). When splitting results by age, those over the age of 65 showed consistent results for both loneliness and anxiety. For those under 65, the association between social disengagement and physical performance was still clearly apparent but all other associations were attenuated (see Supplementary Table 5). When restricting the measures for walking speed and total physical performance to adults aged 60+ (who had provided full walking test data), results were entirely maintained (see Supplementary Table 6). Finally, when applying the SPPB clinical cut-off of < 10 indicating increased risk of disability^26^ and all-cause mortality ^27^, individuals were 10% more likely to be below this threshold if they were socially disengaged isolated and 5% more likely if they were lonely when accounting for all demographic and physical health-related factors. (See Supplementary Table 7).

## Discussion and conclusions

The main finding of this study is that loneliness and aspects of social isolation, including domestic isolation and social disengagement, are independently associated with poorer physical performance in older age. However, low social contact (i.e. low frequency of social contact with family and friends) was not related to physical performance. These relationships were independent of all time-invariant confounders and all identified demographic, physical and mental health-related time-varying confounders and were also broadly consistent across gender. However, for adults under the age of 65, only the results for social disengagement remained. These results are important given that lower SPPB scores have been associated with adverse clinical outcomes including falls^28^, future disability^29^, loss of independence in activities of daily living, rehospitalisation following discharge^30–32^, reductions in mobility and function^31,33^, decline in health status^31^, longer hospital inpatient stay^34^, nursing home admission^21^, and death^21,30^.

As our study was observational rather than interventional, causality cannot be assumed. We have shown a persistent association between social factors and physical performance, and confirmed this is independent of all time-invariant and all identified time-varying confounders. For certain exposures, such as domestic isolation, there is little theoretical rationale for substantial reverse causality (i.e. the causal link between declining physical performance and no longer living with a spouse is less clear than the reverse). However, it is possible that the results for other social factors could be bi-directional, in that lower physical performance may lead to isolation, which may contribute to the findings related to social disengagement. Reductions in physical performance may make social engagement more challenging due to the difficulties experienced getting to the social and cultural events and groups. This may lead to avoidance of these activities resulting in social disengagement. However, it is worth noting that this would still have to be independent of other physical activity as we controlled for time-varying physical activity in our analyses.

These findings are broadly supportive of other studies on this topic, showing that isolation and loneliness are independently associated with physical performance. For example, loneliness and social isolation have previously been shown to be associated with increased rate of motor decline in older adults^14^^,^^15^, and reduced walking speed^16^. However, our results extend these findings in three key ways. First, we used a sophisticated statistical approach that identified that the relationship with physical performance is not just present for static social factors but also for time-varying loneliness and isolation. This suggests that changes in loneliness and isolation rather than just absolute scores at a particular moment in time are related to physical performance. Second, we used a more nuanced categorisation of isolation and showing specific associations between domestic isolation and social disengagement but not frequency of social contact. Third, as our analyses automatically accounted for all time-invariant factors, even if unobserved, these results suggest that although factors such as socio-economic status may explain some of the relationship between loneliness, isolation and physical performance, they do not fully explain the association, as the finding persists independent of these factors.

In considering why some aspects of social isolation, but not frequency of social contact, were related to physical performance, the most obvious explanation is to do with physical activity. Social contact (which included telephone, email and writing as well as face-to-face contact) may not lead to increased physical activity as reliably as social engagement or living with somebody. Indeed, social engagement involves active participation in community activities, which has been proposed to constitute a form of physical activity of adequate intensity to prevent, or at least reduce, deconditioning in physical performance, while individuals who live with somebody have been shown to be more physically active^35–37^. We did also control for inactivity in our analyses, and our sensitivity analyses further controlled for moderate or vigorous sport or physical activity. As the results were not attenuated by the inclusion of such factors, this suggests that while physical activity engaged in as part of social participation may make some contribution to the relationship between social participation and physical performance, it does not fully explain the association. But nonetheless, it is possible that social engagement and living with someone reduces broader sedentary behaviours that are known to lead to increases in physical decline without individuals being consciously aware (and therefore formally reporting) that their activity levels are higher. Alternatively, it is possible that living with somebody and being engaged in community social activities help to provide a sense of purpose greater than that achieved merely through socialising. Purpose and feeling that what one does in life is worthwhile are related, both cross-sectionally and longitudinally, to a wide range of factors that influence physical performance including better mental and physical health (self-rated depressive symptoms and chronic disease), and healthier lifestyles (more physical activity, less smoking, better diet)^38^. An additional potential explanation for the findings regarding isolation is that by physically seeing people, health problems that may impact physical performance are more likely to be brought to the attention of healthcare professionals and receive subsequent intervention or management. This may happen more for face-to-face contact than for email or telephone contact. So, our social isolation variable (which combined face-to-face and technology interactions) may have been a less strong factor in encouraging engagement with health services. Future research is recommended that looks in even greater detail into how different types of face-to-face and remote contact are associated with health outcomes.

It is also of note that we found an association between loneliness and physical performance that was independent of social isolation measures. Much of the literature on loneliness has discussed the potential explanatory role of mental health, due to the association between loneliness and depression^39^. Notably, the strength of our findings was reduced when accounting for depression, and for balance significance was lost, suggesting that mental health does mediate some of the relationship. However, as the association persisted independently for total performance and for walking speed and sit-to-stand, this suggests that there are other independent paths linking loneliness with physical performance. These may be similar to those linking social isolation to physical performance, such as an individual’s sense of purpose, or they may differ. For example, loneliness has been associated with poorer concentration and memory, which could affect physical performance directly or via reduced confidence^40^.

Amongst other limitations not yet mentioned, it is also possible that unmeasured confounding variables may exist that impact or explain the results presented here. Using fixed effects models for our statistical analysis should have reduced the impact of any such un-measured variables, as all time-invariant factors are automatically accounted for even if unobserved. But time-varying unobserved factors remain a challenge. Finally, there may have been an underestimation of changes in SPPB in individuals with high levels of physical functioning, due to a potentially ceiling effect in SPPB for such people. However, this merely suggests that the association presented here may be an underestimation. Future research could consider in more detail how different sub-groups are affected and whether the interaction of risk factors leads to stronger associations between social factors and physical performance.

In conclusion, we have shown that components of isolation and loneliness are independently associated with poorer physical performance in older adults, including worse sit-to-stand ability, balance and slower walking speed. When considering an outcome of physical performance, an important question is whether findings are clinically meaningful. The minimal clinically important difference (MCID) for total SPPB is 0.5 points for a small change and 1 for a substantial change^41^. Therefore, although loneliness and aspects of isolation are associated with poorer physical performance in older age, our findings suggest the degree of this association falls below the MCID. These associations may be of importance on a population scale, but less so on an individual basis. However, the substantially increased risk of falling below the SPPB cut-off of < 10, for socially disengaged or lonely individuals, is of major importance given the implications regarding disability and mortality risk. Arguably, the implication for clinicians and policy makers is that greater attention to social isolation and related factors could be beneficial in designing programmes to improve physical performance. Our findings suggest that societal change, including the increasing reports of isolation and loneliness, may be related to the physical performance of older adults, with further implications for other aspects of health and functioning. Our findings also suggest that physical performance in older adults should be considered in relation to social distancing measures related to the COVID-19 pandemic. These findings do not suggest that social distancing is inappropriate; rather that physical performance interventions that respect the requirements of social distancing measures during this period should be considered. Future intervention studies could explore the impact of social interventions on physical performance to clarify whether this relationship could be causal.

## Supplementary information

Supplementary Tables.

## Data Availability

The Data used in this study are available on application from the UK Data Service (https://www.ukdataservice.ac.uk/).
