# Supplementary material for: Social isolation, loneliness and physical performance in older-adults: fixed effects analyses of a cohort study
Source: Sci Rep. 2020 Aug 17;10:13908. doi: 10.1038/s41598-020-70483-3 (PMC7431531; doi:10.1038/s41598-020-70483-3)
Supplement: Supplementary file 1 — Supplementary Tables. [file 41598_2020_70483_MOESM1_ESM.pdf]

Supplementary Material: Social isolation, loneliness and physical performance in older-adults: fixed effects analyses of a cohort study

Authors: Keir EJ Philip[1,2]*, Michael I Polkey[2], Nicholas S Hopkinson[1,2], Andrew Steptoe[3], Daisy Fancourt[3]

Affiliation/Institution:

1) National Heart and Lung Institute, Imperial College London, London, United Kingdom
2) Respiratory Medicine, Royal Brompton and Harefield NHS Foundation Trust, London, United Kingdom
3) Department of Behavioural Science and Health, University College London, London, United Kingdom

*Corresponding author contact information:

Dr Keir Philip

Address: NHLI Respiratory Muscle Laboratory, Imperial College London, Royal Brompton Campus, Fulham Rd, London SW3 6NP, UK

Email: k.philip@imperial.ac.uk

Tel +44 (0) 20 7351 8029

ORCID iD: https://orcid.org/0000-0001-9614-3580

Supplementary Table1: Social predictors and physical performance across the three time points

**Social predictors**		**Wave 2**	**Wave 4**	**Wave 6**
Domestic isolation	% living alone	26.1%	19.9%	17.7%
Low social contact	Mean score (standard error)<br>(range 0-9; higher indicates more socially isolated)	5.4 (0.02)	5.4 (0.03)	5.4 (0.03)
Social disengagement	Mean score (standard error)<br>(range 2-8; higher indicates more socially disengaged)	5.4 (0.02)	5.3 (0.02)	5.3 (0.02)
Loneliness	Mean score (standard error)<br>(range 3-9; higher indicates more lonely)	4.2 (0.02)	4.2 (0.02)	4.1 (0.02)
**Physical performance**				
Total physical performance	Mean score SPPB (standard error)<br>(range 0-12, higher scores indicate better physical performance)	9.99 (0.03)	9.92 (0.03)	9.93 (0.03)
5 times sit-to-stand	Mean score (standard error)<br>(range 0-4, higher scores indicate better physical performance)	3.03 (0.01)	3.03 (0.01)	3.03 (0.02)
Balance	Mean score (standard error)<br>(range 0-4, higher scores indicate better physical performance)	3.58 (0.01)	3.56 (0.01)	3.55 (0.01)
Walking speed	Mean score (standard error)<br>(range 0-4, higher scores indicate better physical performance)	3.38 (0.01)	3.34 (0.01)	3.34 (0.01)
	**Overall Mean**	**Overall SD**	**Within SD**	**Between SD**
Domestic isolation	0.22	0.41	0.36	0.22
Low social contact	5.42	1.82	1.05	1.53
Social disengagement	5.33	1.75	1.01	1.46
Loneliness	4.19	1.56	1.04	1.17
Total physical performance	9.88	2.42	1.67	1.76
5 times sit-to-stand	3.01	1.10	0.69	0.86
Balance	3.54	0.91	0.57	0.71
Walking speed	3.33	1.02	0.66	0.77

SD=Standard deviation

Supplementary Table 2: Results from fixed effects models showing the relationship between isolation, loneliness and physical performance: each predictor entered in separate models

	Total physical performance		5 times sit-to-stand		Balance		Walking speed	
	Coef (95% CI)	p	Coef (95% CI)	p	Coef (95% CI)	p	Coef (95% CI)	p
Domestic isolation	**-0.35 (-0.49 to -0.22)**	**<.001**	-0.06 (-0.14 to 0.01)	.09	**-0.14 (-0.20 to -0.08)**	**<.001**	**-0.15 (-0.22 to -0.09)**	**<.001**
Low social contact	-0.003 (-0.03 to 0.02)	.678	-0.007 (-0.02 to 0.01)	.37	0.00004 (-0.01 to 0.01)	>.99	0.003 (-0.009 to 0.02)	.59
Social disengagement	**-0.10 (-0.13 to -0.07)**	**<.001**	**-0.02 (-0.04 to -0.01)**	**.002**	**-0.03 (-0.05 to -0.02)**	**<.001**	**-0.04 (-0.06 to -0.03)**	**<.001**
Loneliness	**-0.07 (-0.10 to -0.04)**	**<.001**	**-0.03 (-0.05 to -0.01)**	**.003**	**-0.01 (-0.03 to -0.001)**	**.032**	**-0.03 (-0.04 to -0.01)**	**<.001**

N=8,780, 3 observations per person, total observations 26,340. Fully adjusted model, accounting for all time-invariant factors and time and additionally adjusted for time-varying demographic factors (age, marital status, employment status and wealth), time-varying health factors (BMI, eyesight, comorbidities, chronic pain, frequency of alcohol consumption, smoking habits, inactivity and cognition), and time-varying mental health (depression).

Supplementary Table 3: Results from fixed effects models showing the relationship between isolation, loneliness and physical performance, additionally adjusting for moderate and vigorous exercise/activity

	Total physical performance		5 times sit-to-stand		Balance		Walking speed	
	Coef (95% CI)	p	Coef (95% CI)	p	Coef (95% CI)	p	Coef (95% CI)	p
Domestic isolation	**-0.30 (-0.43 to -0.17)**	**<.001**	-0.04 (-0.11 to 0.03)	.27	**-0.13 (-0.19 to -0.07)**	**<.001**	**-0.13 (-0.19 to -0.07)**	**<.001**
Low social contact	0.003 (-0.02 to 0.03)	.83	-0.004 (-0.02 to 0.01)	.59	0.001 (-0.01 to 0.01)	.85	0.006 (-0.007 to 0.02)	.36
Social disengagement	**-0.08 (-0.11 to -0.05)**	**<.001**	**-0.02 (-0.03 to -0.003)**	**.022**	**-0.03 (-0.04 to -0.02)**	**<.001**	**-0.03 (-0.05 to -0.02)**	**<.001**
Loneliness	**-0.05 (-0.08 to -0.02)**	**.001**	**-0.03 (-0.04 to 0.009)**	**.002**	-0.01 (-0.02 to 0.01)	.25	**-0.02 (-0.03 to -0.01)**	**.002**

N=8,780, 3 observations per person, total observations 26,340. All predictors were entered simultaneously into the models so results are mutually adjusted. All results automatically account for all time-invariant factors and time, and are additionally adjusted for time-varying demographic factors (age, marital status, employment status and wealth), time-varying health factors (BMI, eyesight, comorbidities, chronic pain, frequency of alcohol consumption, smoking habits, inactivity and cognition), time-varying mental health (depression), and time-varying moderate and vigorous physical activity/exercise.

Supplementary Table 4: Results from fixed effects models showing the relationship between isolation, loneliness and physical performance, split by gender

	Total physical performance		5 times sit-to-stand		Balance		Walking speed	
MEN[a]	Coef (95% CI)	p	Coef (95% CI)	p	Coef (95% CI)	p	Coef (95% CI)	p
Domestic isolation	**-0.31 (-0.48 to -0.14)**	**<.001**	-0.05 (-0.15 to 0.04)	.27	**-0.13 (-0.21 to -0.06)**	**.001**	**-0.12 (-0.20 to -0.04)**	**.002**
Low social contact	0.005 (-0.03 to 0.04)	.77	-0.002 (-0.02 to 0.02)	.86	0.001 (-0.01 to 0.01)	.92	0.01 (-0.01 to 0.02)	.46
Social disengagement	**-0.10 (-0.14 to -0.06)**	**<.001**	**-0.02 (-0.04 to -0.003)**	**.02**	**-0.04 (-0.05 to -0.02)**	**<.001**	**-0.04 (-0.06 to -0.03)**	**<.001**

Loneliness	**-0.06 (-0.10 to -0.02)**	**.003**	**-0.03 (-0.05 to -0.01)**	**.008**	-0.01 (-0.03 to 0.01)	.28	**-0.02 (-0.04 to -0.005)**	**.011**
WOMEN[b]	Coef (95% CI)	p	Coef (95% CI)	p	Coef (95% CI)	p	Coef (95% CI)	p
Domestic isolation	**-0.30 (-0.51 to -0.09)**	**.005**	-0.04 (-0.15 to 0.08)	.51	**-0.11 (-0.21 to -0.01)**	**.03**	**-0.15 (-0.25 to -0.05)**	**.005**
Low social contact	-0.003 (-0.04 to 0.03)	.86	-0.008 (-0.03 to 0.01)	.39	0.001 (-0.01 to 0.01)	.92	0.004 (-0.01 to 0.02)	.60
Social disengagement	**-0.09 (-0.13 to -0.05)**	**<.001**	**-0.02 (-0.04 to -0.001)**	**.042**	**-0.03 (-0.05 to -0.01)**	**.002**	**-0.04 (-0.05 to -0.02)**	**<.001**
Loneliness	**-0.05 (-0.10 to -0.004)**	**.033**	-0.02 (-0.05 to 0.0002)	.052	-0.007 (-0.03 to 0.01)	.53	**-0.02 (-0.04 to 0.002)**	**.081**

[a]N=4,831, 3 observations per person, total observations 14,493. [b]N=3,949, 3 observations per person, total observations 11,847. All predictors were entered simultaneously into the models so results are mutually adjusted. All results automatically account for all time-invariant factors and time, and are additionally adjusted for time-varying demographic factors (age, marital status, employment status and wealth), time-varying health factors (BMI, eyesight, comorbidities, chronic pain, frequency of alcohol consumption, smoking habits, inactivity and cognition), time-varying mental health (depression), and time-varying moderate and vigorous physical activity/exercise.

Supplementary Table 5: Results from fixed effects models showing the relationship between isolation, loneliness and physical performance, split by age

	Total physical performance		5 times sit-to-stand		Balance		Walking speed	
50-64[a]	Coef (95% CI)	p	Coef (95% CI)	p	Coef (95% CI)	p	Coef (95% CI)	p
Domestic isolation	-0.16 (-0.37 to 0.04)	.12	-0.04 (-0.16 to 0.07)	.46	-0.06 (-0.15 to 0.03)	.18	-0.05 (-0.15 to 0.04)	.28
Low social contact	0.003 (-0.03 to 0.03)	.84	-0.005 (-0.02 to 0.01)	.54	0.003 (-0.01 to 0.01)	.67	0.01 (-0.01 to 0.02)	.47
Social disengagement	**-0.07 (-0.11 to -0.04)**	**<.001**	-0.02 (-0.03 to 0.004)	.12	**-0.02 (-0.04 to -0.01)**	**.008**	**-0.03 (-0.05 to -0.02)**	**<.001**
Loneliness	**-0.05 (-0.09 to 0.01)**	**.028**	-0.02 (-0.05 to 0.001)	.061	-0.01 (-0.02 to 0.01)	.49	-0.02 (-0.04 to 0.001)	.069
65+[b]	Coef (95% CI)	p	Coef (95% CI)	p	Coef (95% CI)	p	Coef (95% CI)	p
Domestic isolation	**-0.38 (-0.56 to -0.20)**	**<.001**	-0.05 (-0.15 to 0.04)	.28	**-0.16 (-0.24 to -0.08)**	**<.001**	**-0.17 (-0.25 to -0.09)**	**<.001**
Low social contact	0.001 (-0.04 to 0.03)	.98	-0.004 (-0.03 to 0.02)	.69	0.001 (-0.02 to 0.02)	.92	0.005 (-0.01 to 0.02)	.56
Social disengagement	**-0.11 (-0.15 to -0.07)**	**<.001**	**-0.03 (-0.05 to -0.01)**	**.009**	**-0.04 (-0.06 to -0.02)**	**<.001**	**-0.05 (-0.06 to -0.03)**	**<.001**
Loneliness	**-0.06 (-0.10 to -0.02)**	**.007**	**-0.03 (-0.05 to -0.01)**	**.014**	-0.01 (-0.03 to 0.01	.39	**-0.02 (-0.04 to -0.01)**	**.014**

[a]N=4,074, 3 observations per person, total observations 12,222. [b]N=4,706, 3 observations per person, total observations 14,118. All predictors were entered simultaneously into the models so results are mutually adjusted. All results automatically account for all time-invariant factors and time, and are additionally adjusted for time-varying demographic factors (age, marital status, employment status and wealth), time-varying health factors (BMI, eyesight, comorbidities, chronic pain, frequency of alcohol consumption, smoking habits, inactivity and cognition), time-varying mental health (depression), and time-varying moderate and vigorous physical activity/exercise.

Supplementary Table 6: Results from fixed effects models showing the relationship between isolation, loneliness and physical performance only amongst those aged 60+ (when walking speed data was captured).

	Total physical performance		Walking speed	
	Coef (95% CI)	p	Coef (95% CI)	p
Domestic isolation	**-0.37 (-0.53 to -0.21)**	**<.001**	**-0.16 (-0.23 to -0.09)**	**<.001**
Low social contact	0.001 (-0.03 to 0.03)	.96	0.004 (-0.009 to 0.02)	.53
Social disengagement	**-0.10 (-0.14 to -0.07)**	**<.001**	**-0.04 (-0.06 to -0.03)**	**<.001**

Loneliness	**-0.05 (-0.09 to -0.02)**	**.005**	**-0.02 (-0.04 to -0.004)**	**.014**

N=6,183, 3 observations per person, total observations 18,549. All predictors were entered simultaneously into the models so results are mutually adjusted. All results automatically account for all time-invariant factors and time, and are additionally adjusted for time-varying demographic factors (age, marital status, employment status and wealth), time-varying health factors (BMI, eyesight, comorbidities, chronic pain, frequency of alcohol consumption, smoking habits, inactivity and cognition), time-varying mental health (depression), and time-varying moderate and vigorous physical activity/exercise.

Supplementary Table 7: Results from fixed effects models showing the relationship between isolation, loneliness and risk of being below SPPB total score threshold of 10.

	Total physical performance	
	Coef (95% CI)	p
Domestic isolation		
Model 1	**2.89 (2.38 to 3.51)**	**<.001**
Model 2	1.11 (0.89 to 1.39)	.35
Model 3	1.09 (0.87 to 1.38)	.46
Model 4	1.07 (0.85 to 1.35)	.57
Low social contact		
Model 1	1.00 (0.96 to 1.04)	.98
Model 2	1.00 (0.96 to 1.04)	.82
Model 3	0.99 (0.95 to 1.03)	.68
Model 4	0.99 (0.95 to 1.03)	.71
Social disengagement		
Model 1	**1.26 (1.21 to 1.31)**	**<.001**
Model 2	**1.15 (1.10 to 1.20)**	**<.001**
Model 3	**1.10 (1.05 to 1.15)**	**<.001**
Model 4	**1.10 (1.05 to 1.15)**	**<.001**
Loneliness		
Model 1	**1.16 (1.11 to 1.21)**	**<.001**
Model 2	**1.15 (1.10 to 1.21)**	**<.001**
Model 3	**1.10 (1.05 to 1.15)**	**<.001**
Model 4	**1.06 (1.01 to 1.11)**	**.029**

N=4,313 (cases with all positive or all negative outcomes are dropped from analyses), 3 observations per person, total observations 12,939. All predictors were entered simultaneously into the models so results are mutually adjusted. Model 1: accounting for all time-invariant factors and time. Model 2: additionally adjusted for time-varying demographic factors age, marital status, employment status and wealth. Model 3: additionally adjusted for time-varying health factors BMI, eyesight, comorbidities, chronic pain, frequency of alcohol consumption, smoking habits, inactivity and cognition. Model 4: additionally adjusted for time-varying depression.

Supplementary Table 8: Correlations between exposure and outcome measures

	Loneliness	Low social contact	Social disengagement	Domestic isolation	Total physical performance	5 times sit-to-stand	Balance
Low social contact	**0.08**						
Social disengagement	**0.18**	**0.06**					
Domestic isolation	**0.27**	**-0.10**	**0.09**				
Total physical performance	**-0.24**	**0.004**	**-0.32**	**-0.26**			
5 times sit-to-stand	**-0.18**	**-0.01**	**-0.22**	**-0.19**	**0.81**		
Balance	**-0.16**	**0.01**	**-0.24**	**-0.21**	**0.76**	**0.40**	
Walking speed	**-0.23**	**0.01**	**-0.31**	**-0.23**	**0.83**	**0.49**	**0.49**

Boldface indicates significant at p<.001

Supplementary Table 9: Pattern of missing data prior to imputations

Variable	Missing	Total	Percent Missing
Loneliness	8,917	26,340	33.85
Low social contact	12,945	26,340	49.15
Social disengagement	14,373	26,340	54.57
Domestic isolation	0	26,340	0.00
Total physical performance	15,273	26,340	57.98
5 times sit-to-stand	11,945	26,340	45.35
Balance	9,598	26,340	36.44
Walking speed	11,618	26,340	44.11

Age	5,984	26,340	22.72
Employment	6,074	26,340	23.06
Wealth	6,380	26,340	24.22
BMI	8,711	26,340	33.07
Eyesight	5,993	26,340	22.75
Chronic health conditions	6,042	26,340	22.94
Chronic pain	5,984	26,340	22.72
Alcohol consumption	8,922	26,340	33.87
Smoking status	1	26,340	0.00
Inactivity	5,988	26,340	22.73
Cognition	12,545	26,340	47.63
Depression	6,493	26,340	24.65
